# Neoadjuvant chemo-radiotherapy combined with immune checkpoint inhibitors: A case report of rectal small-cell undifferentiated carcinoma achieved pathological complete response

**DOI:** 10.1097/MD.0000000000040368

**Published:** 2024-11-15

**Authors:** Chaoxi Zhou, Linlin Xiao, Fuyin Qu, Ming Liu, Chao Gao, Yi Wang, Yuting Xiao, Yuanhang Gao, Fengpeng Wu, Xuan Wang

**Affiliations:** a Department of Radiation Oncology, The Fourth Hospital of Hebei Medical University, Shijiazhuang, Hebei, China; b Department of General Surgery, Fourth Hospital of Hebei Medical University, Shijiazhuang, Hebei, China.

**Keywords:** case report, immune checkpoint inhibitors, neoadjuvant chemoradiotherapy, pathological complete response, rectal small-cell undifferentiated carcinoma

## Abstract

**Rationale:**

Small-cell undifferentiated carcinoma (SmCC), as an aggressive malignancy, are most commonly arising in lung. Extrapulmonary SmCC is rare. It was reported that SmCC accounts for only 0.1% to 0.2% of colorectal cancers. Currently, no standard treatment regimen is recommended. Here, we presented a case of SmCC from rectum. The patient achieved pathological complete response (pCR) after surgery, which makes us feel gratified, and we are also eager to share this successful case with more peers to provide more references for clinical decision-making.

**Patient concerns:**

A 32-year-old male patient presented himself to our outpatient clinic with defecation difficulty for more than 1 month in November 2021.

**Diagnoses:**

Colonoscopy revealed a rectal mass 4 cm from the anal margin. Pelvic magnetic resonance imaging revealed a mass in the rectal wall, consistent with the appearance of rectal carcinoma. Cancer cell was found after several biopsies and the immunohistochemistry indicated rectal SmCC.

**Interventions:**

Considering that the patient is very young and the malignancy of SmCC is very high, our treatment plan is also very cautious. Many literatures were also searched, but the literature on rectal SmCC is few and the prognosis is poor. Subsequently, we combined the treatment principles of rectal cancer and small cell lung cancer to develop an individualized treatment plan for patients. The patient received neoadjuvant chemoradiotherapy (nCRT) (short-course radiotherapy: 25 Gy/5 fractions, chemotherapy: etoposide + nedaplatin) combined with immune checkpoint inhibitors (ICIs) (tislelizumab). Then, the patient received laparoscopic radical transabdominal resection of rectal carcinoma with a temporary stoma on June 27, 2022.

**Outcomes:**

Postoperative pathology showed that there was chronic inflammation in the rectal mucosa without residual cancer, which meant that the patient achieved pCR after nCRT combined with ICIs. On August 15, 2024, the patient returned to our hospital for review, and no signs of recurrence and metastasis were found. By the time this article is submitted, the patient has survived for more than 35 months.

**Lessons:**

This is the first to be reported in a rectal SmCC patient who achieved pCR after nCRT combined with ICIs, which may provide supporting data for using this treatment option for rectal SmCC.

## 1. Introduction

Small-cell undifferentiated carcinoma (SmCC), as an aggressive malignancy, are most commonly arising in lung. Extra-pulmonary SmCC is rare. It was reported that SmCC accounts for only 0.1% to 0.2% of colorectal cancers.^[1,2]^

At present, the treatment decision basis for SmCC is mainly in the field of small-cell lung cancer (SCLC). Systemic therapy plays a very important role in the treatment of SmCC, even in patients with locally advanced tumor. There seemed to be little change in the chemotherapy of SmCC in the last 20 years, which was still dominated by etoposide and platinum-based drugs. In recent years, chemotherapy combined with immune checkpoint inhibitors (ICIs) has become the preferred treatment option for extensive-stage SCLC.^[3–5]^ In CASPIN study, durvalumab plus EP significantly improved OS versus EP (median OS: 12.9 vs 10.5 months, *P* = .003, 36-month OS: 17.6% vs 5.8%). In Impower 133 study, median OS was 12.3 and 10.3 months (*P* = .0154) with atezolizumab plus CP/ET and placebo plus CP/ET, respectively. For SmCC, radiotherapy (RT) has a potential role in all stages, as part of either definitive or palliative therapy. Radiation oncology input, as part of a multidisciplinary evaluation or discussion, should be provided for all patients early in the determination of the treatment strategy.

For locally advanced rectal carcinoma (RC), neoadjuvant RT combined with fluoropyrimidine-based chemotherapy followed by radical resection is preferred treatment option.^[6]^ Neoadjuvant RT includes long-course chemoradiotherapy (LCRT) and short-course RT (SCRT). ICIs could be considered for patients with mismatch repair-deficient (dMMR)/microsatellite instability-high (MSI-H) disease.^[7–13]^ However, patients with MSI-H rectal tumors is not common in the clinic. The percentage of stage IV colorectal tumors characterized as MSI-H/dMMR ranges from 3.5% to 5.0% in clinical trials and was 6.5% in the Nurses’ Health Study and Health Professionals Follow-up Study.^[14–16]^ Indeed, the NICHE trial demonstrated that ICIs may have a role during early-stage disease in MSS colorectal cancer. The NICHE trial showed 27% pathological responses among patients with early-stage, low TMB, MSS colorectal cancer treated with neoadjuvant nivolumab and ipilimumab combination. Correlative analysis showed that CD8 + PD-1 + cell infiltration was predictive of response.^[17,18]^

Here, we presented a case of SmCC from rectum. For patients with locally advanced RC, neoadjuvant chemoradiotherapy (nCRT) is the preferred treatment, and ICIs is also effective for patients with MMR-proficient (pMMR), especially when combined with hypofractionated radiotherapy. For patients with SmCC, they are very sensitive to radiotherapy and chemotherapy, and ICIs has also achieved excellent efficacy in the treatment of SCLC. In combination with the principles of treatment of SCLC and RC, the treatment strategy we developed for this patient was nCRT combined with ICIs. Ultimately, the patient achieved pathological complete response (pCR) after surgery. We hope that this report could provide supporting data for using this treatment option as one of options for management of rectal SmCC.

## 2. Case report

**Figure 1.**
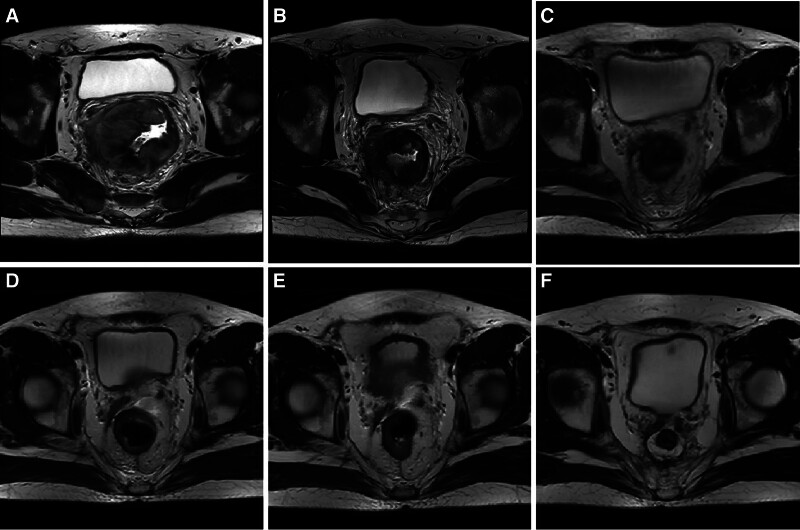
Pelvic magnetic resonance imaging (MRI) image of patients at different stages of treatment. (A) The baseline pelvic MRI image. (B) The pelvic MRI image after short-course radiotherapy and 1 cycle of chemotherapy combined with immune checkpoint inhibitors (ICIs). (C) The pelvic MRI image after 2 cycles of chemotherapy combined with ICIs. (D) The pelvic MRI image after 4 cycles of chemotherapy combined with ICIs. (E) The pelvic MRI image after 6 cycles of chemotherapy combined with ICIs. (F) The pelvic MRI image after surgery.

**Figure 2.**
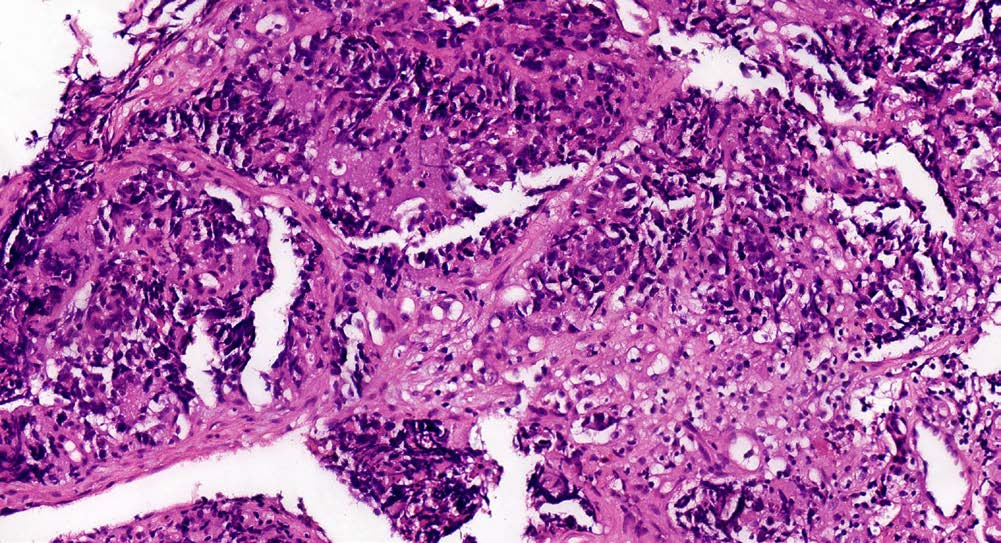
HE staining of the tumor (400× magnification).

**Figure 3.**
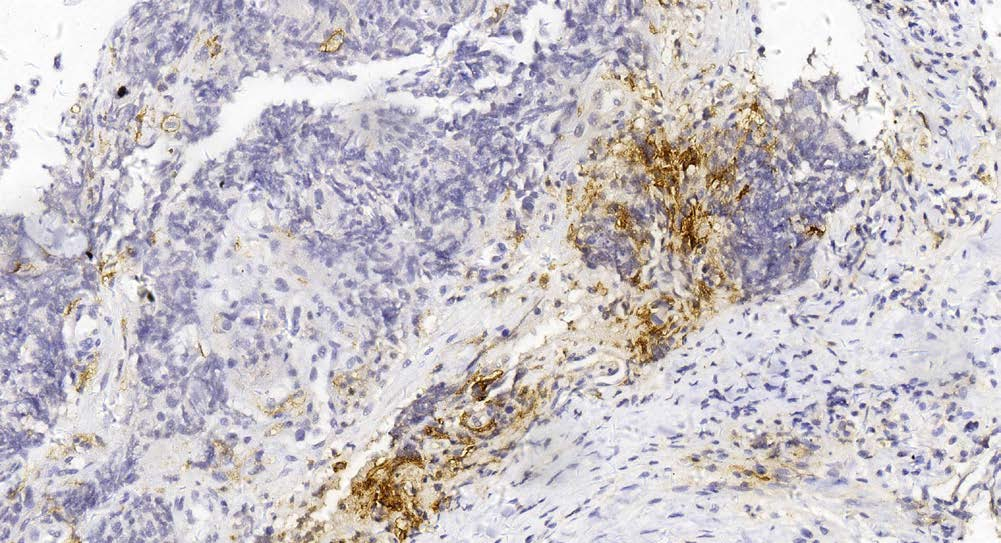
PDL1 staining of the tumor (400× magnification).

A 32-year-old male patient presented himself to our outpatient clinic with defecation difficulty for more than 1 month in November 2021. Colonoscopy revealed a rectal mass 4 cm from the anal margin. Pelvic magnetic resonance imaging (MRI) revealed a mass in the rectal wall, consistent with the appearance of RC (Fig. 1A). The distance between the distal end of the tumor and the anal margin was 8.1 cm, invading 100% of the rectal wall. The sagittal diameter of the tumor was 10.6 cm, and the thickest rectal wall was about 1.5 cm. Cancer cell was found after several biopsies and the immunohistochemistry indicated rectal SmCC (Fig. 2). The combined positive score of programmed cell death ligand 1 was 8 by DAKO 22C3 and 5 by VENTANA SP263 (Fig. 3). The results of MSI and MMR testing were MSI-low (MSI-L) and pMMR. No mutations were detected in RAS and BRAF in this patient. The expression of CD56 was positive. In addition, serum carcinoma embryonic antigen, carbohydrate antigen 19-9, carbohydrate antigen 72-4 determination were all within the normal range, while the neuron-specific enolase determination was 3 times higher than the normal limit. Other urine and stool routine and biochemical tests showed no abnormality. The patient’s baseline examination showed no signs of tumor metastasis.

After multidisciplinary tumor board, the treatment strategy we developed for this patient was nCRT combined with ICIs. The patient was hospitalized and received a course of SCRT to the primary tumor and regional lymph nodes to a dose of 25 Gy in 5 fractions followed by 1 cycle systemic therapy with regimen of etoposide + nedaplatin + tislelizumab (etoposide 100 mg d1–5 + nedapaltin 50 mg d1–3 + tislelizumab 200 mg d1, q21d). Tislelizumab is a humanized IgG4 programmed cell death 1 (PD-1) inhibitors. At the end of this treatment cycle, the patient said that the symptom of defecation difficulty were relieved and pelvic MRI showed that the rectal mass was smaller than before, with a sagittal diameter of 7.2 cm (Fig. 1B). Then the patient continued to receive 5 cycles of consolidation chemotherapy combined with ICIs. The last treatment was performed in May 2022. The patient said that the symptom of defecation difficulty were relieved obviously and pelvic MRI showed that the rectal mass continued to be smaller than before (Fig. 1C–E). Colonoscopy revealed that there was a white scar in the rectum 7 to 10 cm away from the anal edge.

**Figure 4.**
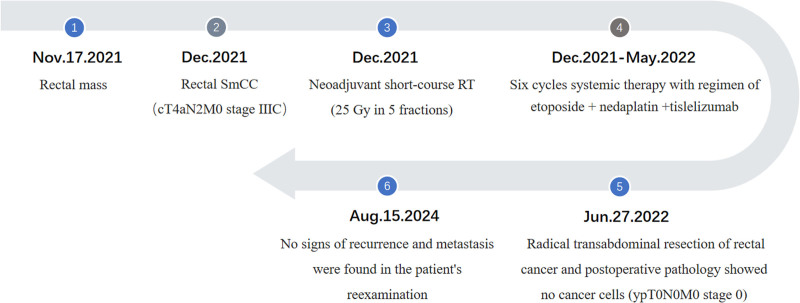
The timeline of the patient’s treatment.

Then, the patient received laparoscopic radical transabdominal resection of RC with a temporary stoma on June 27, 2022. Postoperative pathology showed: there were chronic inflammation and focal lymphocyte infiltration of rectal mucosa. A large amount of foam-like histocytes could be seen from the mucosal layer of the intestinal wall to the outer membrane layer. Local mucous lake was formed and no clear epithelial components were found in the mucosa. All tumors were sampled, and no clear cancer residue was found. Based on the medical history, it was considered that the changes were post-treatment. No cancer cell was found at peritoneal reflexive incisal margin, circumferential incisal margin, and examination (upper incisal margin, lower incisal margin). No metastasis was found in perienteric lymph nodes (0/11). Postoperative pathology showed that there was chronic inflammation in the rectal mucosa without residual cancer, which meant that the patient achieved pCR after nCRT combined with ICIs. On August 15, 2024, the patient returned to our hospital for review, and no signs of recurrence and metastasis were found (Fig. 1F). Figure 4 is the timeline of the treatment. By the time this article is submitted, the patient has survived for more than 35 months.

## 3. Discussion

In this article, we report a case of rectal SmCC, which is uncommon in itself. We found that the patient achieved pCR after nCRT combined with ICIs. By the time this article is submitted, the patient has survived for 35 months. At present, the patient has unobstructed bowel movement, with weight increase of 13 kg.

It is well known that the most common pathological type of rectal cancer is adenocarcinoma, accounting for at least 90%. In addition, there are also rare types of pathology, including SmCC, melanoma, squamous cell carcinoma, lymphoma. Even so, caution should be exercised in the pathological diagnosis owing to the treatment plan varying greatly with different pathological types. In this report, the patient’s laboratory results are different from those of other RC patients. The patient’s serum carcinoma embryonic antigen, carbohydrate antigen 19-9, carbohydrate antigen 72-4 determination were all within the normal range, while the neuron-specific enolase determination was 3 times higher than the normal limit. The patient underwent 2 biopsies but failed. As expected, we finally found the cancer cells in the third biopsy. In the course of pathological diagnosis, the patient’s symptoms of bowel difficulty were so severe that he could hardly eat. In order to relieve the patient’s symptoms, we gave the patient local RT firstly when waiting for the immunohistochemical results. After the final pathological diagnosis was clear, we made the systematic treatment plan for the patient. Fortunately, the patient responded well to the treatment option.

After the nCRT combined with ICIs, the patient said that the symptom of defecation difficulty were relieved obviously and pelvic MRI showed that the rectal mass continued to be smaller than before (Fig. 1C–E). Colonoscopy revealed that there was a white scar in the rectum 7 to 10 cm away from the anal edge. We considered whether to adopt a “wait & watch” strategy. But for several reasons, we finally chose to perform radical surgical resection for the patient in the hope of prolonging the survival time of the patient. First of all, the patient was very young, and the willingness to treat was very positive. Although the imaging and colonoscopy results of the patient after neoadjuvant therapy suggested that clinical complete response (cCR) maybe achieved, cCR is not equivalent to pCR. Second, the pathological type of rectal tumor in the patient was SCC with a high degree of malignancy and a high risk of recurrence and metastasis. So we want to eradicate the tumor as much as possible. Third, the rectal mass of the patient is 8 cm away from the anal margin, and there is no problem of surgical resection to preserve the anus. So we thought the operation will not affect the quality of life of the patient too much.

SmCC of the gastrointestinal (GI) tract is a rare disease. Previous literatures were mostly case reports. Brenner et al summarized 544 patients with SmCC of the GI tract and analyzed the epidemiology, clinical presentation, staging, pathology, etiology, treatment, and prognosis.^[2]^ The prognosis of patients with GI SmCC is very poor. Survival time is about several weeks for patients without treatment and of 6 to 12 months for patients receiving therapy. SmCC tended to behave as a systemic disease instead of a locally advanced tumor. Therefore, treatment should be based on systematic treatment, which usually followed that used in patients with SCLC. Etoposide and cisplatin were the main chemotherapy regiments. ICIs was also playing an increasingly important role in SCLC. Even so, surgery may be the best option to control the primary tumor itself, especially for patients with limited disease. In addition, initial treatment is also very important and it represents the best and presumably the only opportunity to cure limited disease. In this case report, considering that tumor in the patient is limited, the purpose of our treatment plan is to cure it. Hence, we chose neoadjuvant systematic treatment combined with radiotherapy, then surgery was performed after the patient achieved good tumor regression. Eventually the patient achieved pCR, which was very gratifying because the patient was very young.

Neoadjuvant radiotherapy for rectal cancer includes LCRT and SCRT.^[19–26]^ Both types of radiotherapy could reduce patients’ local recurrence rate, but LCRT is more effective in tumor regression. This maybe because that the tumor has not had enough time to regression since surgery was performed 1 week after SCRT. Recently, some researchers found that neoajuvant SCRT followed by chemotherapy yielded higher pCR rates, lower rates of distant metastasis and better survival than neoajuvant LCRT.^[24,25]^ In RAPIDO trial, SCRT followed by 18 weeks of chemotherapy achieved higher pCR rate (28% vs 14%, *P* < .0001) compared with LCRT, which could potentially contribute to organ preservation.^[25]^ In OPRA trial, organ preservation is achievable in half of the patients with RC (3-year TME-free survival: 53%) treated with total neoadjuvant therapy.^[26]^

For patients with MSI-H/dMMR metastatic colorectal cancer, ICIs led to significantly longer survival than chemotherapy when received as first-line therapy.^[7,8,11]^ The final analysis of Keynote 177 confirmed the durable antitumor benefit of pembrolizumab monotherapy with longer progression-free survival (3-year progression-free survival: 42.3% vs 11.1%), higher objective response and complete response, and fewer treatment related adverse events compared with chemotherapy as first-line therapy in patients with MSI-H/dMMR metastatic colorectal cancer.^[11]^ The current Phase II clinical studies results show that ICIs was remarkably effective in dMMR, locally advanced RC.^[9,12,17]^ In study of Cercek A et al, all 12 patients (100%; 95% confidence interval, 74–100) had a cCR by the treatment of single-agent dostarlimab, an anti-PD-1 monoclonal antibody.^[10]^ In the study of Chen G, 17 patients were enrolled and received at least 1 dose of sintilimab, a single-agent PD-1 antibody. The results showed that 6/16 (37.5%) patients underwent surgery, of whom 3 (18.8%) patients had a pCR, 9/16 (56.3%) patients had a cCR and chose the “wait & watch” strategy.^[12]^ In the study of Chalabi M et al, all the 20 patients with dMMR tumors, who received combination treatment with ipilimumab + nivolumab, had a pathological response. Nineteen (95%) patients had an MPR with ≤ 10% residual viable tumor, and this included 12 (60%) pCRs. Furthermore, 4/15 (27%) patients with pMMR tumors showed a pathological response.^[17]^ In addition, some studies had shown that hypofractionated radiotherapy may activate the immune response, and neoajuvant SCRT combined with ICIs may further improve patients’ pCR rate and survival.^[27,28]^ For patients with rectal SmCC, patients who received radiotherapy had a more significant survival benefit than those who did not.^[29]^ In this case report, the neoadjuvant therapy was SCRT, followed by consolidation chemotherapy combined with ICIs. Finally, the patient’s tumor retreated completely.

There were several potential constraints and challenges in the management of this patient. Firstly, the patient is very young and the malignancy of SmCC is very high. Currently, no standard treatment regimen is recommended. Secondly, pathological acquisition of the patient went through a tortuous process. No cancer cells were found until a third biopsy was performed, which brought great trouble to the subsequent treatment. Thirdly, the patient’s symptoms of bowel difficulty were so severe that he could hardly eat. However, because there was no pathology, we could not give the patient an appropriate systemic treatment plan, only local radiotherapy to relieve symptoms. Fourthly, many literatures were also searched, but the literature on rectal SmCC is few and the prognosis is poor. Finally, we combined the treatment principles of rectal cancer and SCLC to develop an individualized treatment plan for patients. Delightingly, the patient achieved pCR after surgery, which makes us feel gratified, meanwhile, we are also eager to share this successful case with more peers to provide more references for clinical decision-making.

Due to the low incidence of rectal SmCC, it is difficult to conduct clinical trials in these patients. This is the first to be reported in a rectal SmCC patient who achieved pCR after nCRT combined with ICIs, which will add to the existing publications and provide supporting data for this treatment option of rectal SmCC. In conclusion, radical treatment is recommended for limited disease of rectal SmCC. In developing an individualized treatment plan for patients, a comprehensive reference is made to the principles of SCLC and rectal cancer.

## 4. Patient’ perspective

In November 2021, I had my first visit with severe dysostosis. I was only 32 years old, and the initial test results suggested that I had a rectal malignancy. The subsequent further examination gave me a greater blow, because I learned from the doctor that I had a rare and highly malignant rectal tumor, and the prognosis was worse than the general rectal cancer. My attending doctor made an individualized treatment plan for me. Although I expected that the effect might not be good, I was still young and willing to cooperate with the treatment actively. After the initial chemoradiotherapy, I has a significantly alleviate the symptoms of defecation difficulties and regularly review also suggests the tumor been narrowed. My tolerance of the whole treatment process was also acceptable, and I also relinked the hope of survival. I actively cooperated with the relevant antitumor treatment before the operation.

I eventually had surgery to remove it, and fortunately, my doctor told me that there were no cancer cells left in my surgical specimen. During the follow-up of more than 2 years after surgery, my condition has been very stable, with no signs of recurrence or metastasis. I am very grateful to my doctor for making this very good treatment plan for me, and I also agree with the doctor to show my treatment process to more doctors and patients.

## Author contributions

**Conceptualization:** Chaoxi Zhou, Linlin Xiao, Chao Gao, Yuting Xiao, Yuanhang Gao, Fengpeng Wu, Xuan Wang.

**Data curation:** Linlin Xiao, Yi Wang, Yuting Xiao, Yuanhang Gao, Fengpeng Wu, Xuan Wang.

**Formal analysis:** Chaoxi Zhou, Yi Wang.

**Funding acquisition:** Linlin Xiao.

**Investigation:** Chaoxi Zhou, Fuyin Qu, Yi Wang.

**Methodology:** Fuyin Qu, Ming Liu, Yi Wang.

**Project administration:** Chaoxi Zhou, Fuyin Qu, Ming Liu, Chao Gao, Yuting Xiao, Yuanhang Gao.

**Resources:** Linlin Xiao, Ming Liu, Yuting Xiao, Xuan Wang.

**Software:** Fuyin Qu.

**Supervision:** Yuanhang Gao.

**Validation:** Ming Liu, Chao Gao, Yuanhang Gao.

**Visualization:** Chao Gao, Yuting Xiao, Fengpeng Wu.

**Writing – original draft:** Linlin Xiao, Fengpeng Wu, Xuan Wang.

**Writing – review & editing:** Fengpeng Wu.
